# Animal laboratory training improves lung ultrasound proficiency and speed

**DOI:** 10.1186/1757-7241-21-S1-S5

**Published:** 2013-05-28

**Authors:** NP Oveland, HM Lossius, R Aagaard, J Connolly, E Sloth, L Knudsen

**Affiliations:** 1 Department of Research and Development, Norwegian Air Ambulance Foundation, Droebak, Norway; 2 Department of Anesthesiology and Intensive Care, Stavanger University Hospital, Stavanger, Norway; 3 Department of Surgical Sciences, University of Bergen, Bergen, Norway; 4 Faculty of Health Sciences, Institute of Clinical Medicine, Aarhus University, Aarhus, Denmark; 5 Emergency Department, Royal Victoria Infirmary, Newcastle upon Tyne, UK; 6 Department of Anesthesiology and Intensive Care, Aarhus University Hospital, Aarhus, Denmark

## Background

Although lung ultrasound (US) is accurate in diagnosing pneumothorax (PTX), the training requirements and methods necessary to perform US examinations must be defined.

## Study objective

To test whether animal laboratory training (ALT) improves the diagnostic competency and speed of PTX detection with US.

## Methods

Twenty medical students without US experience attended a 1-day course. Didactic, practical and experimental lectures covered basic of US physics, US machines and lung US, followed by hands-on training to demonstrate the signs of normal lung sliding and PTX. Each student’s diagnostic skill level was tested with three subsequent examinations (day 1, day 2 and a 6-month follow-up) using experimentally induced PTX in porcine models. The outcome measures were sensitivity and specificity for US detection of PTX, self-reported diagnostic confidence and scan time.

## Results

The students improved their skills between the initial two examinations: sensitivity from 81.7% (69.1-90.1) to 100.0% (94.3-100.0), and specificity from 90.0% (82.0-94.8) to 98.9% (92.3-100.0); these improvements were sustained 6 months later. There was a significant positive learning curve in choosing the correct answers (p=0.018), a 1-point increase in the self-reported diagnostic confidence (7.8 to 8.8 on a 10-point scale; p<0.05) and a 1-minute reduction in the mean scan time per lung (p<0.05).

## Conclusion

Without previous experience and after undergoing training in an animal laboratory, medical students improved their diagnostic proficiency and speed for PTX detection with US. Lung US is a basic technique with a steep learning curve and may be used by multiple medical specialties to accurately diagnose PTX.

